# Functional strengthening through synaptic scaling upon connectivity disruption in neuronal cultures

**DOI:** 10.1162/netn_a_00156

**Published:** 2020-12-01

**Authors:** Estefanía Estévez-Priego, Sara Teller, Clara Granell, Alex Arenas, Jordi Soriano

**Affiliations:** Departament de Física de la Matèria Condensada, Universitat de Barcelona, Barcelona, Spain; Universitat de Barcelona Institute of Complex Systems (UBICS), Barcelona, Spain; Departament de Física de la Matèria Condensada, Universitat de Barcelona, Barcelona, Spain; Universitat de Barcelona Institute of Complex Systems (UBICS), Barcelona, Spain; GOTHAM Lab – Institute for Biocomputation and Physics of Complex Systems (BIFI), University of Zaragoza, Zaragoza, Spain; Department of Condensed Matter Physics, University of Zaragoza, Zaragoza, Spain; Departament d’Enginyeria Informàtica i Matemàtiques, Universitat Rovira i Virgili, Tarragona, Spain; Departament de Física de la Matèria Condensada, Universitat de Barcelona, Barcelona, Spain; Universitat de Barcelona Institute of Complex Systems (UBICS), Barcelona, Spain

**Keywords:** Neuronal cultures, Calcium imaging, CNQX, Effective connectivity, Global efficiency, Functional organization, Synaptic scaling

## Abstract

An elusive phenomenon in network neuroscience is the extent of neuronal activity remodeling upon damage. Here, we investigate the action of gradual synaptic blockade on the effective connectivity in cortical networks in vitro. We use two neuronal cultures configurations—one formed by about 130 neuronal aggregates and another one formed by about 600 individual neurons—and monitor their spontaneous activity upon progressive weakening of excitatory connectivity. We report that the effective connectivity in all cultures exhibits a first phase of transient strengthening followed by a second phase of steady deterioration. We quantify these phases by measuring G_EFF_, the global efficiency in processing network information. We term *hyperefficiency* the sudden strengthening of G_EFF_ upon network deterioration, which increases by 20–50% depending on culture type. Relying on numerical simulations we reveal the role of *synaptic scaling*, an activity–dependent mechanism for synaptic plasticity, in counteracting the perturbative action, neatly reproducing the observed hyperefficiency. Our results demonstrate the importance of synaptic scaling as resilience mechanism.

## Author Summary

Neuronal circuits exhibit homeostatic plasticity mechanisms to cope with perturbations or damage. A central mechanism is ‘synaptic scaling,’ a self-organized response in which the strength of neurons’ excitatory synapses is adjusted to compensate for activity variations. Here we present experiments in which the excitatory connectivity of in vitro cortical networks is progressively weakened through chemical action. The spontaneous activity and effective connectivity of the whole network is monitored as degradation progresses, and the capacity of the network for broad information communication is quantified through the global efficiency. We observed that the network responded to the perturbation by strengthening the effective connectivity, reaching a hyperefficient state for moderate perturbations. The study proves the importance of ‘synaptic scaling’ as a driver for functional reorganization and network-wide resilience.

## INTRODUCTION

Response to perturbations or damage in living neuronal circuits is a central yet unresolved problem in neuroscience. A simple network description of damage intuitively suggests that the failure of connectivity pathways precipitates a progressive deterioration of network activity that ultimately compromises the functionality of the system. Realistic descriptions of living neuronal circuits, however, expose the complex interplay between the physical architecture of the network, neuronal dynamics, and plasticity mechanisms in governing circuit behavior, framing scenarios in which response to damage is not only possible, but fast and networkwide. This is particularly important in the context of brain and neurological disorders, since adequate prognosis of functional alterations may help to elucidate actions to stop or revert damage. Studies in stroke, for instance, have pointed out the important role of plasticity in preventing a cascade of degradation and fostering recovery (Calabresi, Centonze, Pisani, Cupini, & Bernardi, 2003; Murphy & Corbett, 2009). Current multidisciplinary approaches for prognosis are making an effort to integrate acquired experimental data with numerical models to characterize neuronal damage and subsequent network responses (Bassett & Sporns, 2017; Fornito, Zalesky, & Breakspear, 2015). Thus, understanding the different actors at play—connectivity, dynamics, and plasticity—and their interrelation has become a central goal in medical network neuroscience.

In the last few years, substantial efforts have been invested in linking structural characteristics of neuronal networks with their capacity to cope with perturbations and damage (Aerts, Fias, Caeyenberghs, & Marinazzo, 2016; Farooq, Chen, Georgiou, Tannenbaum, & Lenglet, 2019; Majdandzic et al., 2013). Specifically for brain functional networks, studies unveiled the importance of modular and hierarchical organizations (Meunier, Lambiotte, Fornito, Ersche, & Bullmore, 2009), hubs (van den Heuvel & Sporns, 2013), and other node structural aspects (Bassett & Sporns, 2017; Bassett, Zurn, & Gold, 2018; Sporns, 2014; Stam, 2014). Despite the progress and enlightenment in this direction, the incorporation of plasticity and self-regulatory mechanisms as additional actors is opening new avenues for understanding resilience to insult. Indeed, the brain and other living neuronal circuits are reliant on diverse adaptive mechanisms for proper performance and stability that include synaptic plasticity and structural plasticity (Butz, Steenbuck, & van Ooyen, 2014; Fauth & Tetzlaff, 2016; Fauth, Wörgötter, & Tetzlaff, 2015). These mechanisms are central to regain circuits’ operability upon severe perturbations or injury (Barral & Reyes, 2016; Costa, Mizusaki, Sjöström, & van Rossum, 2017; Marder, 2011; Murphy & Corbett, 2009; Teller et al., 2019). One of the most important synaptic plasticity mechanisms is synaptic scaling, in which the strength of excitatory synapses is adjusted to compensate for variations in activity (Desai, Rutherford, & Turrigiano, 1999; Fong, Newman, Potter, & Wenner, 2015; G. G. Turrigiano, Leslie, Desai, Rutherford, & Nelson, 1998). The capacity of synaptic scaling to regulate neuronal activity was demonstrated both in vivo (Echegoyen, Neu, Graber, & Soltesz, 2007; G. Turrigiano, 2012; G. G. Turrigiano, 2008) and in vitro (Barral & Reyes, 2016; Hanes et al., 2020) at a synapse level. Although different investigations pointed out the role of synaptic scaling in correcting neuronal activity after damage, for instance in relation to Alzheimer’s patients cognitive deficits (Borge-Holthoefer, Moreno, & Arenas, 2011) and retinal lesions (Keck et al., 2013), there are no studies demonstrating its impact in the functional organization of a neuronal circuit.

The aim of this study is to analyze the action of synaptic scaling from a network neuroscience perspective and expose its potential as a resilience mechanism. To this end, we designed an experimental pipeline in which small cortical circuits in vitro were exposed to a malfunction-like event that gradually weakened the connectivity among interconnected neuronal assemblies (*clusters*), and analyzed simultaneously the activity of the entire network. The perturbative action was delivered through increasing doses of CNQX, an AMPA-glutamate receptor antagonist in excitatory synapses (Soriano, Martínez, Tlusty, & Moses, 2008; Tibau, Valencia, & Soriano, 2013a). Although CNQX targeted synaptic connectivity, we measured solely its impact on the spontaneous activity, that is, the spontaneous dynamic interactions among clusters. Functional characteristics of the cultured networks were therefore quantified by measuring the alterations in activity as the blockade progressed and by computing the *global efficiency* (Latora & Marchiori, 2001; Rubinov & Sporns, 2010), a measure of the integration of the flow of information in a networked system. Our experiments showed that, as CNQX was applied, there was an initial phase in which the global efficiency raised, revealing an increased information flow capacity in the network. This hyperefficiency was reproducible among network realizations, although the level of impact depended on culture details. Beyond this phase, global efficiency progressively decayed until activity ceased. By using numerical simulations we showed that synaptic scaling, simulated as a local strengthening of remaining effective connections upon damage, was sufficient to explain the experimental behavior. Simple models based on bond percolation (Kirkpatrick, 1973), that is, the degradation or deletion of connections without any scaling, failed at capturing this hyperefficiency phenomenon. To our knowledge, the experiments and modeling presented here is the first strong evidence of the impact of synaptic scaling in preserving network efficiency and whole-system performance, thus exposing synaptic scaling as an intrinsic mechanism for resilience in living neuronal networks. Our study strengthens the vision of synaptic scaling as an emergent and general property of living neuronal circuits, and paves the way toward a better prediction of the action of perturbations and damage in such circuits.

## RESULTS

### Weakening of Synaptic Communication Alters Functional Organization

**Figure 1.**
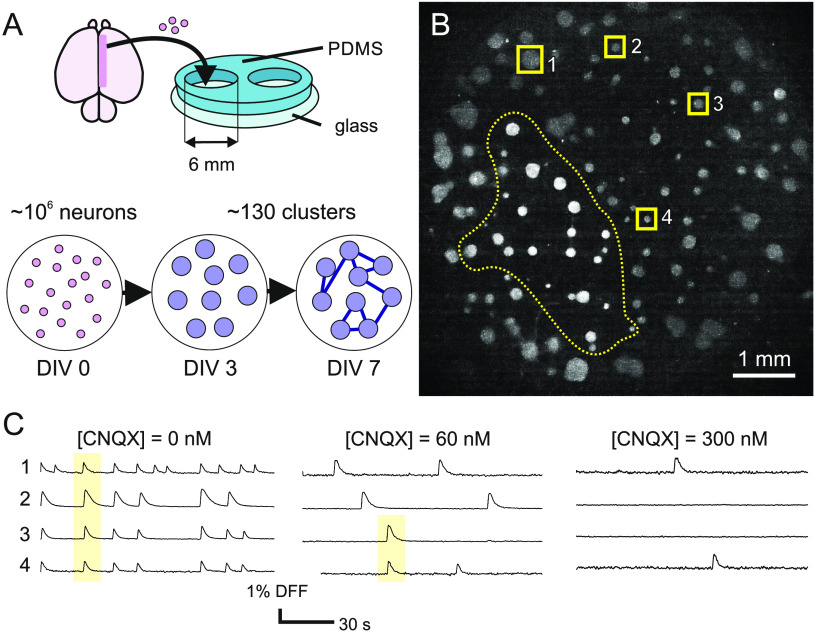
Clustered neuronal cultures and experimental procedure. (A) Schematic preparation of clustered neuronal networks. Dissociated rat cortical neurons were plated on a 6-mm-diameter PDMS wells. The absence of adhesive proteins facilitated neuronal aggregation, giving rise to an assembly of neuronal islands (*clusters*) by *day in vitro* (DIV7). (B) Fluorescence image of a typical neuronal culture. Circular objects are neuronal clusters. Labels highlight four the location of four clusters whose fluorescence traces are shown in panel C. (C) Representative fluorescence traces of four clusters for gradually higher levels of the AMPA glutamate receptor antagonist CNQX. Sharp increases of the signal correspond to activity events. Yellow boxes illustrate episodes of coordinated activity. The overall activity of the clusters decreases with CNQX as a result of the reduced excitatory drive. DFF indicates normalized fluorescence signal, given by DFF(%) = 100 · (*F* − *F*_0_)/*F* − 0, with *F*_0_ the fluorescence signal at rest.

We investigated spontaneous activity in clustered neuronal cultures prepared in 6-mm-diameter PDMS wells on glass, as illustrated in Figure 1A. These cultures emerged as a self-organized process in which the absence of adhesive proteins in the glass substrate favored neuronal mobility and aggregation (Segev, Benveniste, Shapira, & Ben-Jacob, 2003; Teller et al., 2014; Teller, Tahirbegi, Mir, Samitier, & Soriano, 2015). By *day in vitro* (DIV) 7 upon preparation, aggregation shaped highly compact neuronal islands termed *clusters* that remained stable in position and size. In our experiments, about 130 neuronal clusters typically formed in the 6-mm-diameter wells (Figure 1B). Table 1 summarizes the set of networks investigated and their characteristics. The spontaneous activity in the clustered networks was monitored at DIV 8–12 through calcium fluorescence imaging (Figure 1B), which allowed us to follow the evolution of all clusters in the culture with high spatial and temporal resolution. Active clusters appeared as bright objects whose fluorescence intensity ramped up upon activation. The advantage of clustered neuronal cultures is that they exhibit spontaneous activity with a rich spatiotemporal structure (Teller et al., 2014, 2015), in which clusters coactivate in groups of varying size and timing (Figure 1B–C). Since the structure of coactivation patterns, and in turn network functional characteristics, is grounded on the coupling among clusters, these cultures stand out as a particularly suited experimental preparation to investigate the impact of chemical perturbations or damage (Teller et al., 2014, 2015, 2019). The richly structured dynamics of clustered networks—and sister designs in the form of engineered circuits (Kanner et al., 2015; Yamamoto et al., 2018)—is enlightening since dynamics shape networks with a functional organization qualitatively similar to that of the brain, with modular traits and hubs (Teller et al., 2015; Yamamoto et al., 2018). Thus, our clustered cultures combine the controllability and accessibility of an in vitro system with some of the dynamical richness and functional complexity of naturally formed neuronal circuits.

**Table 1.** Summary of the structural and dynamical characteristics of the eight clustered networks investigated.

**Experiment**	**DIV**	***N***	***ϕ* (μm)**	**d (μm)**	***a* (bursts/min)**	〈*k*〉	〈*w*〉
1	8	157	156 ± 56	406 ± 16	7.10	6.0 ± 3.1	2.8 ± 0.5
2	8	125	170 ± 55	437 ± 15	2.52	5.9 ± 2.8	3.2 ± 0.6
3	8	116	177 ± 60	455 ± 14	1.47	6.3 ± 2.7	2.9 ± 0.5
4	9	152	137 ± 34	282 ± 8	5.51	5.3 ± 2.1	3.0 ± 0.4
5	9	144	177 ± 53	411 ± 15	1.71	4.4 ± 1.9	3.3 ± 0.7
6	9	124	160 ± 52	452 ± 15	7.14	5.0 ± 1.7	2.9 ± 0.6
7	9	118	215 ± 68	469 ± 18	3.85	4.9 ± 1.9	3.1 ± 1.1
8	12	128	193 ± 70	462 ± 15	2.91	5.7 ± 2.2	2.9 ± 0.5

*Note*. DIV is the day *in vitro* of the culture, *N* the number of clusters, *ϕ* their average diameter, *d* the typical clusters’ interdistance, *a* the average collective activity, 〈*k*〉 the average effective connectivity per cluster, and 〈*w*〉 the average weight of effective connections. *ϕ*, *d*, 〈*k*〉, and 〈*w*〉 are reported as the means ± standard deviations.

In our experiments we were interested in analyzing the dynamics of a clustered network as the connectivity strength among clusters, and in turn their dynamic coupling, was reduced. The ability of our experimental system to simultaneously monitor the entire network made it unique to investigate the action of perturbations and their impact on network functional organization. Since spontaneous coordinated activity is mostly mediated by the excitatory drive of AMPA-glutamate receptors, both the capacity of the network to fire and cluster-to-cluster synaptic coupling could be altered in a controlled manner through the blockade of AMPA receptors. Thus, following Soriano, Rodríguez Martínez, Tlusty, and Moses (2008), we targeted the excitatory AMPA-glutamate receptors with gradually higher doses of the antagonist CNQX, causing a progressive reduction of spontaneous activity until it ceased. For clarity in the analysis of the data and its interpretation, NMDA excitatory receptors (which account for about 20% of the total excitatory drive in cortical neurons) and GABA inhibitory receptors were fully blocked with the corresponding antagonists.

An example of the impact of CNQX on network spontaneous activity is shown in Figure 1C. Here, the fluorescence traces of four representative clusters are depicted for gradually higher CNQX levels. Without blockade, all four clusters exhibited a rich activity with strong coordination. As CNQX was applied, the level of activity and coordination swiftly decreased, and for full synaptic blockade the clusters either fired weakly and independently or became silent.

**Figure 2.**
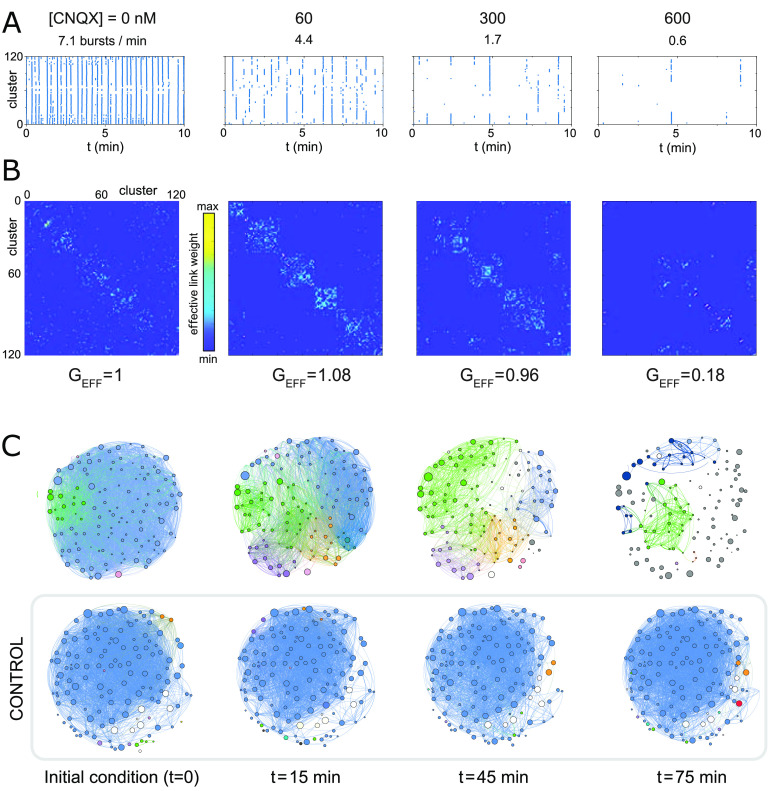
Network activity and functional organization upon degradation. (A) Representative raster plots of spontaneous activity for gradually higher excitatory blockade. Blue dots mark the clusters’ activations. The concentration of CNQX and the average collective spontaneous activity *a* are indicated above each panel. Rasters are limited to 10 min for clarity. (B) Corresponding effective connectivity matrices. The brighter the color, the higher the connectivity weight among two clusters. Matrices are ordered according to the functional communities at [CNQX] = 60 nM. The scaled global efficiency G_EFF_ increases at [CNQX] = 60 nM to steadily decrease afterwards. (C) Effective connectivity maps of perturbed and control experiments. Clusters’ and links’ colors identify different functional communities. Control experiments experienced the same experimental pipeline as perturbed ones, but only GABA_A_ and NMDA receptors were blocked. No significant alterations in the effective connectivity were observed. The perturbed experiment corresponds to the data shown in (A).

The fall of spontaneous activity and the breakdown of clusters’ coactivation patterns for the entire network are shown as raster plots in Figure 2A. In the plots, the dots mark the timing of clusters’ activation, data that are directly obtained from the analysis of the fluorescence signal. In the sequence of raster plots, [CNQX] = 0 (unperturbed network) shapes a strongly coherent dynamics in which most of the clusters coactivate at unison, a feature that reflects the strong coupling among clusters. The strength of spontaneous activity was quantified through the *collective activitya*, defined as the frequency of activity episodes (*bursts*) in which at least five clusters participated (about 5% of the network). Activity *a* was high for [CNQX] = 0, with clusters exhibiting about 7 bursts/min. Activity severely dropped to about 4.4 bursts/min for the small perturbation with [CNQX] = 60 nM, which was accompanied by a strong disruption of the unison behavior, rendering an overall dynamic in which clusters activated in groups of varying size. The decay in activity accentuated as CNQX grew, although some groups of clusters still maintained strong coordinated activity even for relatively large doses of [CNQX] = 600 nM. Spontaneous activity finally stopped by [CNQX] ≳ 1,000 nM. In our study we investigated a total of eight clustered networks. All of them exhibited a similar qualitative trend, although the structure of coordinated activity and its changes on degradation depended on the cluster-to-cluster wiring details in the culture (Table 1).

Activity data was next analyzed in detail to compute the effective connectivity of the networks, allowing us to quantify whole-network alterations on CNQX action. The effective networks (weighted and directed) were extracted using transfer entropy, an approach that we showed appropriate for neuronal cultures in previous studies (Stetter, Battaglia, Soriano, & Geisel, 2012; Teller et al., 2014; Tibau, Ludl, Rüdiger, Orlandi, & Soriano, 2020). From the inferred effective networks we extracted the *global efficiency* G_EFF_, which informs about the capability of the network to operate as a whole. For clarity, the global efficiency was always scaled relative to its value in the unperturbed condition. We note that the global efficiency is based on the calculation of the shortest topological paths among connected nodes in a network, and that for weighted networks the shorter paths are those with stronger weight. Thus, an increase in G_EFF_ can arise from either a higher number of links, stronger weights, or both.

Figure 2B provides an example of the inferred effective connectivity matrices. A summary of the average connectivity 〈*k*〉 and average weight 〈*w*〉 for the eight studied cultures is provided in Table 1. Functional communities were already present for [CNQX] = 0, a feature that indicates that the observed coordinated activity was shaped by strongly interacting groups of clusters. This is clear in the corresponding connectivity map of Figure 2C, in which the majority of the clusters belonged to the same community, as calculated by modularity optimization using the Louvain algorithm (Blondel, Guillaume, Lambiotte, & Lefebvre, 2008). Well-defined communities emerged for [CNQX] = 60 nM and higher concentrations, as indicated by the richer structure of the connectivity maps, which show gradually smaller and more isolated communities. For [CNQX] ≳ 600 nM activity practically ceased and no functional characteristics could be rendered.

The connectivity maps of Figure 2C also helped to picture the CNQX degradation process in terms of the spatial arrangement of the clusters. The maps showed that the functional communities were physically compact, that is, they were constituted by groups of adjacent clusters. This indicates that the clusters typically connected to their immediate neighbors upon network formation, and suggests that the overall dynamics of the network are mediated by local interactions. In this direction, the maps also show that the physical location of the functional communities was preserved, but that they reduced in size as CNQX grew.

### Emergence of Hyperefficiency in CNQX-Perturbed Networks

The above gradual fragmentation of the network with CNQX was not accompanied by a steady loss of clusters’ intercommunication. Surprisingly, for the moderate CNQX concentration of 60 nM, the global efficiency G_EFF_ increased by 8%—for the particular experiment of Figure 2—with respect to the initial condition, to later fall as expected. We note that the increase in G_EFF_ strongly contrasts with the fall of spontaneous activity, which is reduced by 60%. We term *hyperefficiency* this abrupt increase in G_EFF_ and, as we elaborate later, its presence indicates the nonlinear relationship between the structural alterations caused by CNQX and the effective connectivity ones that emerge from dynamical interactions among the clusters. In other words, while CNQX action on excitatory synapses is steady and follows a percolation-like silencing process (Soriano, Rodríguez Martínez, et al., 2008), the effective connectivity obeys more complex mechanisms in which switching dynamic interactions and plasticity-driven adaptation play a central role.

Before investigating the possible hyperefficiency mechanisms, we verified that this phenomenon was not an experimental artifact by comparing the evolution of CNQX-dosed networks with control ones that were prepared and manipulated identically. Figure 2C compares the connectivity maps of the representative perturbed network with a control one. Along the experiment, which lasted 75 min, the control culture retained the same functional traits across the network with small variations, while the degraded one significantly changed.

The data of the representative experiment of Figure 2 was a general trend that consistently repeated across eight different cultures, despite their variability in number of clusters, spontaneous activity, and effective connectivity traits. In all cases, CNQX caused a fragmentation of the network into communities together with a sudden increase of G_EFF_ for moderate CNQX levels.

**Figure 3.**
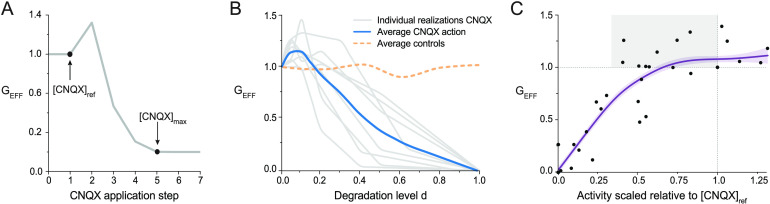
Treatment of experimental curves and relationship between spontaneous activity and global efficiency. (A) Definition of [CNQX]_ref_ (concentration before any network alteration occurred) and [CNQX]_max_ (no network activity). (B) Alignment of the eight experiments (gray curves), their average (blue), and comparison with four averaged controls (orange). (C) Evolution of G_EFF_ as a function of the average global activity *a* for every CNQX concentration. Data is scaled relative to [CNQX]_ref_ to pool all experiments together and highlight deviations from the unperturbed condition (dotted lines). The plot shows that hyperefficiency states (G_EFF_ > 1) are compatible with low spontaneous activity rates (gray box). The thick blue line is a nonlinear scatter plot smoothing to indicate the general trend of the data. Its shading is the confidence interval set as 80%.

To average over culture realizations, however, we observed that each culture responded slightly differently upon perturbation. This resulted in specific CNQX concentrations for the beginning of functional alterations and the end of activity. Thus, for each culture, we considered a *reference* value [CNQX]_ref_ above which dynamic alterations were detectable, and a *maximum* [CNQX]_max_ at which activity fully ceased (Figure 3A). Data was then rescaled according to these points to obtain the global efficiency as a function of an adequate control parameter that we term *degradation leveld*. The degradation level *d* = 0 means that no CNQX is applied and therefore the culture exhibits its natural dynamics, while *d* = 1 corresponds to the CNQX dose that is able to fully silence the culture’s activity. The resulting G_EFF_(*d*) curves for the eight experiments and their average are shown Figure 3B. All analyzed cultures exhibited hyperefficiency, which ranged from a minimum of 3% to a maximum of 50%, and that on average procured about 20% G_EFF_ increase. This clear peak in global efficiency was inexistent in controls.

An aspect that is important to point out is the complex relationship between clusters’ collective activity *a* and global efficiency G_EFF_. The data of Figure 2A and B shows that the hyperefficiency observed at [CNQX] = 60 nM coincides with a prominent decay in network’s collective activity. The reason for this apparent contradiction is that the global activity just informs about the frequency of collective behavior, but does not capture the local dynamic interaction among clusters. This information is provided by the effective connectivity, from which the global efficiency is computed. Thus, we hypothesize that the clusters strengthen their coupling despite coactivating together less often. The complex relationship between *a* and G_EFF_ is illustrated in Figure 3C, in which we plotted one against the other for each CNQX concentration, but scaled the values relative to [CNQX]_ref_ to pool all eight experiments in a single plot. Interestingly, several of the points corresponding to a drop in activity, for example, in the range 0.5–1, are associated to G_EFF_ values above 1 (shaded area). These points are precisely the ones that are behind the observed hyperefficiency, and that we associate to a strengthening of the effective coupling among clusters. This analysis thus suggests that a drop in global activity caused by CNQX is responded to by an increase in clusters’ effective coupling.

### A Degradation Model with Synaptic Scaling Reproduces the Hyperefficiency Phenomenon

**Figure 4.**
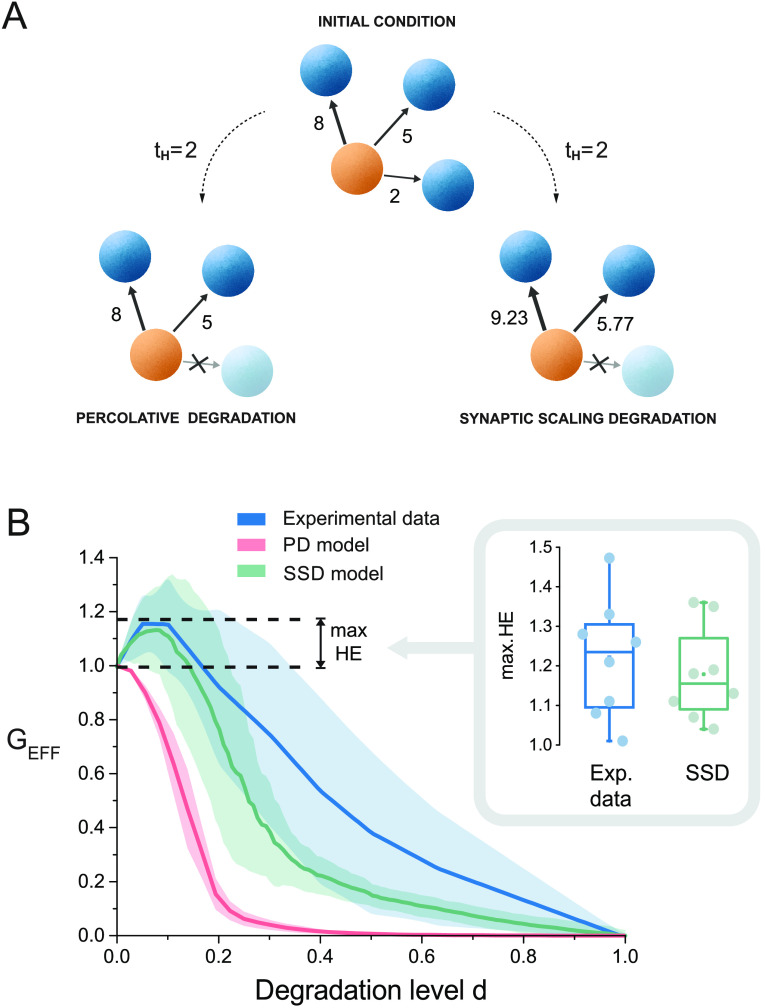
Numerical models and hyperefficiency. (A) Sketch of the models used to simulate the impact of a gradual loss of effective connections. In this example, the two models target an effective connection with weight *t*_*H*_ = 2. For percolative degradation (PD, left) the bottom connection is simply removed upon attack. For synaptic scaling degradation (SSD, right) the same connection is removed, but the surviving outgoing connections are upscaled to distribute the lost weight proportionally among theirs. All curves are averages over eight realizations, and shadings indicate standard deviation. (B) Comparison of the degradation action between experiments (blue) and numerical models (PD, pink; SSD, green). PD portrays a fast-decaying behavior and the network becomes disconnected by *d* ≃ 0.4. SSD reproduces the hyperefficiency (HE) peak and the gentle decay of G_EFF_ with *d* up to the end of the disintegration process. The inset shows the distribution of maximum hyperefficiency values for experiments and model. Despite the dispersion in the data, SSD procured overall similar values.

We carried out numerical simulations to understand the origin of hyperefficiency in the CNQX perturbed experiments. In the simulations we considered the same effective networks as in the experiments for [CNQX]_ref_ and applied two connectivity degradation schemes, namely *percolative degradation* (PD) and *synaptic scaling degradation* (SSD). As sketched in Figure 4A, on the one hand, PD corresponds to a standard bond percolation in which links are gradually removed from weaker to stronger according to a threshold *t*_*H*_. This model reflects the situation in which clusters’ dynamic interaction progressively vanish as degradation grows. On the other hand, SSD consists in the same link removal but with the crucial inclusion of synaptic scaling, which is simulated as the strengthening of the surviving links’ weights proportionally to the lost link’s weight. This model captures a compensatory response mechanism for the loss in activity. In both models, the thresholds are recalculated after the removal process to target the next weakest link, yielding a naturally increasing sequence of thresholds. To compare the models with the experiments, the thresholds *t*_*H*_ were rescaled in the range [0,1] to concord with the degradation level *d*.

The outcome of the two models, and their comparison with experimental data, is shown in the main plot of Figure 4B. PD led to a fast decay of G_EFF_ and a total loss of network connectivity for *d* ≃ 0.4, a fast degradation that clearly is not present in the experiments. On the contrary, SSD not only captured the experimental hyperefficiency for low *d* (inset of Figure 4B), but portrayed a slow decay of G_EFF_ on *d* for strong blockade that mimicked the experimental trend. However, the SSD curve was always below the experimental one. We ascribe this discrepancy to the coexistence of different plasticity mechanisms in the experimental networks, which could give rise to new interactions between previously disconnected clusters, shape new effective connections, and increase G_EFF_. Thus, based on these considerations, we argue that synaptic scaling suffices to explain hyperefficiency but only in the context of weak damage, relative to the percentage of affected links, and not to strong blockade where different plasticity mechanisms can simultaneously emerge.

### Altered Functional Connectivity After Washout Suggests CNQX-Activated Plasticity

**Figure 5.**
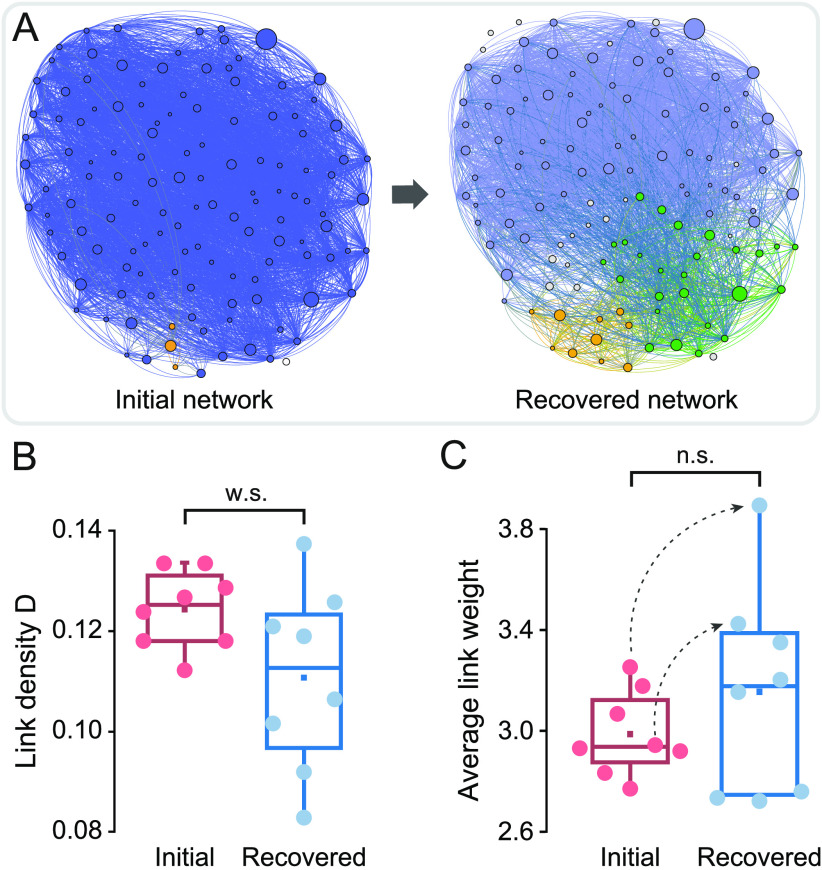
Network recovery after washing off CNQX. (A) Illustrative effective connectivity maps at the beginning of the experiment (left) and after CNQX washout (right). A new community appeared at the bottom of the map for the latter. (B) Comparison of the distribution of link densities *D* between the initial and recovered conditions; w.s.: weak significance, *p* = 0.073 (Student’s *t* test). (C) Corresponding comparison of the average weight of effective connections. The recovered networks exhibited a trend toward higher weights, with two particular experiments boosting up by 20% (dotted curves). Distributions were not significantly different (n.s., *p* = 0.301, Student’s *t* test).

To examine whether CNQX application caused irreversible alterations in the studied networks, we investigated network effective connectivity after recovering the biochemical conditions of the initial network, that is, after washing off CNQX. As a general trend, all investigated neuronal cultures recovered from the perturbation, illustrating the reversible nature of the chemical blockade as opposed to a much more aggressive, and irrecoverable, physical damage. The functionality of the circuits, however, slightly varied despite recovery. As shown in Figure 5A, the effective connectivity for a representative network showed an increase in the number of functional communities. Since the effective connectivity reflects activity, the change in the network functional organization hints at a local strengthening and reorganization of intercluster coupling and dynamics. We must note that all clusters remained alive and active, indicating that no irreversible damage was caused in the network upon CNQX application.

To quantify the extent of effective connectivity changes after washing off CNQX, we computed the density of links *D*, defined as the ratio between the existing effective links (regardless of their weight) and all possible links that can be formed in the network. *D* was computed for each analyzed culture, and then the distribution of *D* values for the eight cultures was plotted to compare the initial and recovered (washout) scenarios. As shown in Figure 5B, the recovered networks exhibited a broader distribution with a trend toward smaller values of *D*. Statistical analysis indicated a weakly significant difference (*p* = 0.073, Student’s *t* test), but the overall trend indicates that intercluster interactions were reduced. On the other hand, the distributions of average weights 〈*w*〉 (Figure 5C) exhibited an opposite trend, with a tendency for the distributions of weights after washout to broaden and the weight values to increase as compared to the initial state. Although no statistical significance was observed (*p* = 0.301, Student’s *t* test), two cultures substantially increased their average weights by about 20%, indicating a strengthening of clusters’ coactivation patterns. Altogether, we conjecture that the incapacity of the network to activate for high CNQX values fostered plastic mechanisms and strong remodeling in an attempt to recover activity, which translated into an altered functional organization at washout.

### Hyperefficiency is Also Observed in Homogeneous Neuronal Cultures

**Figure 6.**
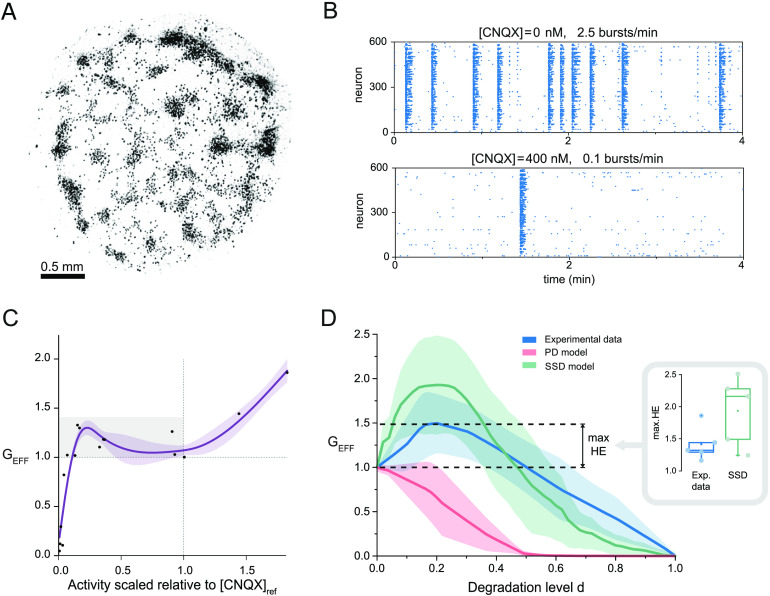
Experiments on homogeneous networks. (A) Highly contrasted bright-field image of a network formed by ≈ 600 individual neurons spread over a substrate. Dark dots are single neurons. (B) Representative raster plots of spontaneous activity for [CNQX] = 0 (top) and 400 nM (bottom). Activity substantially dropped upon CNQX application but coherent activity was preserved. (C) Relationship between scaled global spontaneous activity and G_EFF_ for every CNQX concentration. Data are scaled relative to [CNQX]_ref_ to pool all experiments together and highlight deviations from the unperturbed condition (dotted lines). The gray box highlights those data points with low *a* and G_EFF_ > 1. The thick blue line is a nonlinear scatter plot smoothing, with its shading the confidence interval set as 80%. (D) G_EFF_(*d*) curves for homogeneous experiments (blue) and their corresponding PD (pink) and SSD simulations (green). Data is an average over four realizations. Shadings indicate standard deviation. The inset shows the distribution of maximum hyperefficiency (HE) values between experiments and SSD model.

As a final investigation, we carried out experiments and repeated the analysis in a different configuration of in vitro networks formed by individual neurons grown on a glass coverslip (Figure 6A). These networks are often termed ‘homogeneous’ since single neuronal bodies can be tracked (Tibau, Valencia, & Soriano, 2013b; Tibau et al., 2020). However, despite the uniform distribution of neurons upon preparation, they tend to show some aggregation, although not as extreme as the clustered cultures. An important aspect of homogeneous networks is that they exhibit a characteristically extreme bursting behavior in which all neurons activate together or remain practically silent (Tibau et al., 2013b), completely lacking the rich variability of coactivation patterns observed in the clustered cultures. Representative raster plots of spontaneous activity in these homogeneous cultures are shown in Figure 6B for two CNQX doses, and illustrate that CNQX reduced the frequency of bursting but maintained the collective coherent activity.

In total we studied four homogeneous cultures and analyzed them identically as before. Figure 6C shows the relationship between G_EFF_ and the scaled global activity *a*. Again, we observed that low levels of activity coexisted with values G_EFF_ > 1, indicating the capacity of the neurons to strengthen their dynamical coupling within the bursting events. The evolution of G_EFF_ as a function of *d* is depicted in Figure 6D. Experimental results show a clear hyperefficiency that is also reproduced by the SSD simulations of the experimental networks, although the simulations show stronger peaks (inset of Figure 6D). As before, we ascribe these differences to the coexistence of different compensatory mechanisms in the homogeneous cultures.

## DISCUSSION

In this study we investigated the action of an antagonist of excitatory receptors, CNQX, on the activity and functional organization of neuronal cultures. In our approach, the connectivity among neurons was gradually weakened but the physical connections were left intact. Progressive application of CNQX induced a gradual reduction of synaptic excitatory coupling that caused a drop in the network’s average spontaneous activity. Surprisingly, this progressive degradation was not accompanied by a fall of network information flow, but by a sudden increase of it. Two different types of neuronal cultures were explored, termed *clustered* and *homogeneous*, and both exhibited the same boost in network communication for moderate network degradation. We used the global efficiency G_EFF_ as a measure of information flow capacity across the network for different degradation levels *d*, and quantified a transient yet noticeable G_EFF_ hyperefficiency, a feature that can be viewed as an enhancement of broad network communication as a response to connectivity degradation. To our knowledge, the experimental results shown here are the first strong evidence of enhanced network-wide global efficiency upon connectivity and activity degradation in living neuronal circuits.

We ascribe the transient nature of hyperefficiency to the constraints in the number of physical connections and their strength, with clustered and homogeneous cultures portraying different G_EFF_(*d*) behaviors. The former showed an average hyperefficiency of about 20% and a relatively fast decay, while the latter showed an average hyperefficiency of about 50% and a slower decay. These differences reflect the importance of the structural connectivity in shaping adaptation and remodeling, aspects of great importance (Fauth & Tetzlaff, 2016; Fauth et al., 2015), and that neuronal cultures offer an invaluable scenario to investigate in detail. Although the experimental exploration of the impact of structural connectivity on hyperefficiency is a substantial endeavor beyond the scope of this work, recent advances in neuroengineering suggest that the approach is feasible. In a recent study, Yamamoto et al. (2018) designed networks of four intercoupled modules, and observed that the global efficiency of the network could swiftly change by small variations in the intermodule functional connectivity mediated through CNQX.

Experiments were conducted by applying incremental doses of CNQX and analyzing the network’s effective connectivity at each CNQX step. This progressive degradation maximized our capacity to detect alterations, since the adaptation of the network to a given CNQX concentration was followed in few minutes by the application of a higher concentration. Thus, the cultures were in a state of continuous adaptation. We indeed observed that, in some particular experiments, the structure of collective activity changed during the 15-min recording at a preset CNQX concentration, with some clusters increasing activity or coactivating in larger groups toward the end of it. We thus hypothesize that an experimental pipeline of random CNQX application (instead of progressive) would possibly reduce the degree of hyperefficiency or make it unnoticeable. Additionally, an intriguing open question of our experimental procedure is whether the altered effective connectivity at the peak of hyperefficiency could be maintained by just keeping the cultures in the preset CNQX concentration, that is, without perturbing them any longer. Since the hyperefficiency was observed at relatively low CNQX concentrations in the range 30–120 nM, we conjecture that the ability of neuronal cultures to regulate themselves toward a ‘set point’ of optimal activity (Slomowitz et al., 2015) would gradually return cultures’ activity and global efficiency altered states toward the ones before the perturbation in a few hours.

The recovered networks after washout of CNQX showed important effective connectivity alterations that reveal strong functional remodeling. The density of effective links decreased while the average links’ weights increased, suggesting that information flow was rerouted toward a lower number of paths, but that those were stronger. Such a functional remodeling was also reported in recent experiments in which physical damage was applied to clustered cultures similar to ours (Teller et al., 2019). It was observed that remodeling occurred in a similar way, that is, with the strengthening of available effective links, and in time scales on the order of minutes. Thus, in the context of our study, it is clear that synaptic scaling acts fast and facilitates a continuous adaptation to alterations. Additionally, the strong effective remodeling upon CNQX application is a well-known example of homeostatic plasticity in the literature. Neuronal cultures that have been fully silenced for about 2 days with saturating levels of CNQX exhibit, after washout, a substantially elevated spontaneous activity state as a consequence of the homeostatic strengthening of excitatory synaptic connectivity (G. G. Turrigiano & Nelson, 2004). It was also observed that cultures gradually returned to pre–CNQX synaptic levels in about 36 h (O’Brien et al., 1998). We therefore hypothesize that our cultures would have gradually returned to their initial, pre–CNQX state in a timescale of hours after the final washout.

A wealth of experimental data in neuronal networks supports the swift activation of homeostatic plasticity mechanisms either to maintain stable levels of activity in a fluctuating environment or to provide a corrective response to sudden alterations (Barral & Reyes, 2016; G. Turrigiano, 2012; G. G. Turrigiano & Nelson, 2004). In our experiments, the time spanned between the application of CNQX and a corresponding increase in the global efficiency was on the order of minutes, indicating that the plasticity mechanisms behind the observed hyperefficiency acted fast. We thus hypothesized that short-term synaptic scaling was the dominant actor in governing network behavior. The two numerical models that we introduced, namely percolative degradation (PD) and synaptic scaling degradation (SSD), provided a playground to test this hypothesis. PD served as a reference scenario to demonstrate that the absence of plasticity was incompatible with the experimental observations. The SSD model incorporated a simple form of synaptic scaling in which available effective links were strengthened to compensate for the decay in activity. Such a scaling facilitated the maintenance of information flow across the network since a core of effective connections were always available for communication. The good agreement between experiments and SSD model indicates that synaptic scaling is the dominant plasticity effect. The model could be improved in a number of ways. A first one could be the incorporation of additional plasticity rules, such as ‘Hebbian’ mechanisms of long-term potentiation or long-term depression (G. G. Turrigiano & Nelson, 2004) that would help to refine the agreement between experiments and model. And a second, and more important one, could be the design of detailed simulations that incorporated different culture-like structural blueprints (such as clustered or homogeneous networks) (Hernández-Navarro, Orlandi, Cerruti, Vives, & Soriano, 2017; Orlandi, Soriano, Alvarez-Lacalle, Teller, & Casademunt, 2013; Tibau et al., 2020), neuronal dynamics, and structural plasticity (Fauth & Tetzlaff, 2016; Fauth et al., 2015), thus procuring a complete numerical package to understand the experimental behavior. However, despite the substantial room for improvement of the modeling approach, our aim here was to demonstrate the important role of synaptic scaling in counterbalancing activity loss and shaping large-scale network functionality.

Our study shows that plastic mechanisms are continuously at play in neuronal networks. Different experimental investigations have pointed out the tendency of neuronal circuits to maintain a target level of activity, and our study illustrates that the response to a perturbation may trigger substantial changes at functional levels. This is important in the context of studies that model perturbations and damage purely from the point of view of the distribution of connections. Network characteristics may help to shape overall behavior of living neuronal circuits, but are not sufficient to quantify their response and evolution in a changing environment. Thus, our study not only proves that plasticity is central, but exposes its predictive power when properly incorporated into network models, being able to successfully forecast an accentuation or decay in the dynamics and communicability traits of the network. Our work is thus important for the scientific community that develops models of damage and recovery in complex networks. Degradation with synaptic scaling, as defined along the manuscript, corresponds to an elusive percolation process, whose analytical details have still to be revealed.

Our findings enhance the idea that the brain can react to pathological perturbations by using compensation mechanisms, striving to maintain a homeostatic performance. The hyperefficiency shown here has similarities with the hyperpriming phenomena observed in cognitive studies in Alzheimer’s patients (Borge-Holthoefer et al., 2011). The consequences of damaging the system and its subsequent recovery serve to simulate pathological scenarios. The study of alterations when a controlled and reproducible disturbance is applied, aims to find patterns amidst a given event and the reaction it causes, to determine vulnerabilities and strengths in respect to disease and dysfunction.

## METHODS

### Clustered Neuronal Cultures

Neuronal cortical cultures were prepared from Sprague-Dawley rat embryonic brains at 18 to 19 days of development. As described previously (Teller et al., 2014, 2015), dissection was carried out in ice-cold L-15 medium, cortical tissue dissociated by repeated pipetting, and neurons plated on twin 6-mm-diameter polydimethylsiloxane (PDMS) cavities attached to a glass coverslip (Figure 1A). The diameter of the cavities was chosen to coincide with the field of view of the imaging system and that allowed the visualization of the entire neuronal network. Cultures were incubated at 37°C, 5% CO_2_, and 95% humidity in cell culture medium. At *day in vitro* (DIV) 5 cultures were treated with 0.5% FUDR and Uridine to restrict glial proliferation. Afterward, the cells were maintained by replacing the culture medium every 3 days.

The absence of adhesive proteins in the glass coverslips facilitated aggregation of neurons and glia, giving rise to compact assemblies termed *clusters* (Shein Idelson, Ben-Jacob, & Hanein, 2011; Teller et al., 2014) by DIV 5. Each PDMS cavity typically encompassed about 130 clusters that were uniformly spread on the surface of the glass (Figure 1B). Cluster’s size and physical arrangement were stable between DIV 7 and 18.

A total of eight clustered neuronal cultures were investigated. The cluster’s spatial characteristics for each culture are provided in Table 1 and include the number of clusters *N*; the average diameter *ϕ*, measured over bright field images of the cultures; and the typical distance *d* between clusters, measured as the average Euclidean distance between a cluster and its four closest neighbors. The low standard deviation of *d* is a good indicator of the uniformity in clusters’ spatial arrangement. Variability among clusters’ diameters was broad, in the range 100–250 μm, leading to a number of neurons in the range 60–380 (Shein Idelson et al., 2011), but no differences could be observed in clusters’ dynamics despite their differences in number of neurons (Teller et al., 2014).

### Homogeneous Neuronal Cultures

Homogeneous neural cultures were prepared by seeding dissociated neurons on 3-mm PDMS wells whose surface was previously coated with the adhesive protein poly-L-lisine (PLL), and following the same procedures as described by Tibau et al. (2020). Some aggregation still occurred in these cultures despite the reduced mobility of the neurons due to PLL (Figure 6A), but about 600 individual neurons could be identified and their dynamics monitored. All experiments with homogeneous cultures were carried at DIV 12.

### Calcium Fluorescence Imaging

Spontaneous activity in either clustered or homogeneous cultures was monitored through fluorescence calcium imaging (Teller et al., 2014, 2015; Tibau et al., 2020), which allows the detection of calcium transients during neuronal activations as sharp increases in the fluorescence signal (Figure 1C). Before recording, the culture of interest was incubated in darkness for 20 min in a pH stable recording solution (RS) that contained 1.5 μg/ml of the green calcium fluorescence probe Fluo 8 AM. The culture was then transferred to a glass bottom petri dish filled with 2 ml of fresh RS.

Recordings were conducted at room temperature on a Zeiss Axiovert inverted microscope equipped with a high-speed camera (Hamamatsu Orca Flash 4.0) in combination with a fluorescence light source. Images were acquired with the camera software Hokawo 2.10 at a rate of 50 frames per second (fps), a size of 1,024 × 1,024 pixels, and a spatial resolution of 5.76 μm/pixel.

### Spontaneous Activity and Network Bursts

Clustered cultures were measured at DIV 8–12, a developmental stage in which the clusters exhibited spontaneous activity with at least 1 collective activation (*burst*) per minute (Table 1). Since, as observed previously by Teller et al. (2014, 2015), spontaneous activity in clustered cultures is often of a modular nature, in which the size of collective activations encompasses from few clusters to the entire network, we considered as burst those activity events in which at least five clusters coactivated in a window of 200 ms (Teller et al., 2014).

Homgeneous cultures were measured at DIV 12 and exhibited strongly coherent dynamics, in which the whole network activated together during a bursting episode, remaining practically silent in between bursts. Average bursting frequency in the four studied cultures was 9.6 ± 4.9 bursts/min.

### Experimental Procedure and Pharmacology

Both excitatory and inhibitory connections were present in the clustered cultures. To investigate the changes in activity induced solely by progressively weaker AMPA glutamate excitation in neurons, NMDA excitatory receptors, which account for 20% of excitatory drive in cortical neurons, were blocked with 20 μM of the receptor antagonist APV (Sigma A5282), and GABA_A_ inhibitory receptors were blocked with 40 μM of the antagonist bicuculline (Sigma B7561). Both antagonists were applied simultaneously and the culture was left 5 min in darkness for biochemical stabilization. A 15-min recording was then conducted and swiftly analyzed to evaluate the initial state of the network. Cultures exhibiting poor or fragmented activity were discarded. For the highly active cultures, AMPA glutamate excitatory connectivity was then gradually weakened by application of the receptor antagonist CNQX, with preset concentrations of [CNQX] (nM) = 30,60,120,300,600, 1,000. For each CNQX concentration, spontaneous activity was recorded for 15 min. Cultures were kept in darkness for 5 min in between recordings for the next drug application to take effect.

To investigate whether the neuronal cultures were able to recover the activity patterns prior to CNQX action, the studied clustered cultures were washed off with fresh RS at the end of the recording session, APV and bicuculine applied again, and activity monitored for additional 20 min.

Control experiments were carried out to verify that the entire procedure and duration of the recordings did not affect the health of the neurons. Control recordings consisted identical manipulations as standard experiments but without addition of CNQX.

A practically identical procedure was followed with homogeneous cultures, with the only difference that the CNQX concentrations were adjusted following Tibau et al. (2020) and Tibau et al. (2013b), and used [CNQX] (nM) = 100, 200, 400, 800, 1,000, 2,000.

### Data Analysis

#### Fluorescence traces.

For each experiment, neuronal clusters or individual neurons were manually identified on a highly contrasted fluorescence image and ascribed as regions of interest (ROIs). The average fluorescence (gray scale level) within each ROI was then computed, and the raw fluorescence traces *F*_*i*_(*t*) for each cluster *i* extracted. Each trace was then corrected from global drifts and artifacts and normalized as DFF_*i*_(%) ≡ 100 ⋅ (*F*_*i*_(*t*) − *F*_*i*,0_)/*F*_*i*,0_, where *F*_*i*,0_ if the basal fluorescence of each cluster. The onset times of activation of clusters or individual neurons were determined as those events in which the fluorescence trace crossed a preset threshold, set as the mean + 2 times the standard deviation of the entire fluorescence trace (Teller et al., 2014, 2015). Trains of activity were then built by ascribing as ‘1’ the existence of an activation at a given time point, and ‘0’ otherwise.

### Network Analysis

#### Effective connectivity.

It was inferred using a modified version of transfer entropy (TE) (Schreiber, 2000). TE was computed for each pair of nodes *X* and *Y* (either clusters or neurons) with signals *x*_*m*_ and *y*_*m*_ indexed by 0 ≤ *m* ≤ *m*_max_, where *m*_max_ is the total number of time steps in the data, as

$${\text{TE}}_{Y\to X}=-\underset{\begin{array}{c} 0\leq n\leq n_{\text{max}}\\ 0\leq i\leq i_{M} \end{array}}{\sum }p\left(x_{m+1},x_{m}^{\left(i\right)},y_{m}^{\left(i\right)}\right)\times \log_{2}\frac{p\left(x_{m+1}|x_{m}^{\left(i\right)},y_{m}^{\left(i\right)}\right)}{p\left(x_{m+1}|x_{m}^{\left(i\right)}\right)},$$ (1)

where *i* is the index of the past time step considered, that is, the length of the vectors {$x_{m}^{\left(i\right)}$}, and *i*_*M*_ = 2 is the Markov order of the model. Here, instantaneous feedback was assumed, meaning that *X* and *Y* could interact within a time bin, as in generalized transfer entropy (Orlandi, Stetter, Soriano, Geisel, & Battaglia, 2014; Stetter et al., 2012). Effective connectivity was inferred for the 15-min-long raster plots (*m*_max_ = 45,000 points with an acquisition rate of 50 fps). TE = 0 for an identical or random pair of signals. Since experimental recordings are short, it may occur that random activations in nearby time bins are taken as causal interactions. Thus, to reject spurious connections, significance *z* was established for any connection *X* to *Y* by comparing the TE_*Y*→*X*_ estimate with the joint distribution of TE for all input scores *X*′ to *Y* and output scores *X* to *Y*′ (for any *X*′ and *Y*′), as

$$z=\frac{{\text{TE}}_{Y\to X}-\langle {\text{TE}}_{\text{joint}}\rangle }{\sigma_{\text{joint}}},$$ (2)

where 〈TE_joint_〉 is the average value of the joint distribution and *σ*_joint_ is its standard deviation. Significant connections were then set as those with *z* ≥ 2. This threshold was considered optimal since it captured the flow of neuronal communication during activity at both local and global scales. A lower threshold of *z* = 1 yielded networks that excessively emphasized whole-network coordinated activity, effectively shaping random graphs in all studied cases. Thresholds *z* ≳ 3 emphasized the strongest neuron-to-neuron interactions only and often yielded empty matrices. All results presented here were consistent with thresholds in the range 1.5 ≤ *z* ≤ 2.5. By construction, the adjacency matrix **A** = {*a*_*ij*_} of significant connections was directed and weighted, conceptually capturing the strength of dynamic interactions between clusters or neurons, and with the weight of the connection given in standard deviation units of the joint distribution.

*Density of links D*. For a network with *N* nodes, it was defined as the fraction of total existing effective links (regardless their weight) to all possible links, that is, *D* = ∑_*ij*_ *b*_*ij*_/(*N*(*N* − 1)), where **B** = {*b*_*ij*_} is the binarized adjacency matrix of **A**.

*Single realization global efficiency G*. The analysis of the data for each investigated culture provided a set effective adjacency matrices {*a*_*ij*_}_*k*_, where *k* is the step of CNQX application. For each of these matrices, the global efficiency *G* of a network with *N* nodes was determined following the standard definition (Latora & Marchiori, 2001; Rubinov & Sporns, 2010)

$$G=\frac{1}{N(N-1)}\underset{i\neq j}{\sum }\frac{1}{d(i,j)},$$ (3)

where *d*(*i*, *j*) is the shortest weighted path calculated on the matrix {*a*_*ij*_}, that is, the smallest sum of weights throughout all possible paths between *i* and *j*. In this construction, higher connection weights correspond to shorter lengths, and therefore the global efficiency increases with the weight of the connections. If no path exists between nodes *i* and *j* then *d*(*i*, *j*) = ∞, so that there is no contribution to the summation in Equation 3. The term *N*(*N* − 1) in this equation is a normalization factor that accounts for all possible directed links that can be formed by the *N* nodes. Global efficiency was calculated using the Brain Connectivity Toolbox in Matlab.

### Degradation Level *d* and Alignment of Experimental Curves

The CNQX concentrations were converted into a well-defined parameter that captured in a range [0, 1] the progressive damage of a neuronal network. Thus, for each experiment *n*, the CNQX concentrations were transformed into steps of a *degradation level d* by defining

$$d_{k}^{\left(n\right)}=\frac{{\text{[CNQX]}}_{k}^{\left(n\right)}-{\text{[CNQX]}}_{\text{ref}}^{\left(n\right)}}{{\text{[CNQX]}}_{\max}^{\left(n\right)}},$$ (4)

where *k* is the step of CNQX application along the experiment, [CNQX]_*k*_ the corresponding concentration value, [CNQX]_ref_ the reference concentration above which the dynamic alterations in the network are measurable, and [CNQX]_max_ the concentration that fully silenced the network. With this construction, the degradation level *d* varied between 0 (no alteration to the network) to 1 (full silencing of activity and complete effective network breakdown). Data outside these bounds was disregarded. Since [CNQX]_ref_ and [CNQX]_max_ varied across cultures, the use of *d* provided a robust manner to average among cultures.

### Scaled Global Efficiency G_EFF_ and Averaged G_EFF_(*d*) Curves

The set of global efficiency values for each experiment, *G*^(*n*)^(*d*), was scaled with respect to its *reference* value prior damage, as G_EFF_^(*n*)^(*d*) = *G*^(*n*)^(*d*)/*G*_ref_^(*n*)^, where *G*_ref_^(*n*)^ is the global efficiency measured at *d* = 0. The G_EFF_^(*n*)^(*d*) curves were then interpolated as 100 equidistant points by using a cubic interpolation that preserved the shape of the curves, and averaged over *n* = 8 experimental realizations (clustered cultures) or *n* = 4 (homogeneous cultures).

### Numerical Models for Network Degradation

Two numerical models were proposed to elucidate the impact of gradual degradation on network global efficiency. Both models considered the same initial effective networks as in the experiments for *d* = 0, denoted as {$a_{ij}^{\left(0\right)}$}, and applied a series of rules for the deletion of links. Since the effective connectivity matrices were obtained from transfer entropy with a significance threshold *z* = 2, the values {$a_{ij}^{\left(0\right)}$} were bounded between *a*_min_ = 2 and a given *a*_max_ that depended on each network.

*Percolative degradation* (PD). This model progressively deleted the effective output links whose weights were equal or lower than a threshold *t*_*H*_, starting at *t*_*H*_ = *a*_min_ and progressively targeting stronger links (Figure 4A, left). Thus, at a given simulation step, the deleted links were those that fulfilled *t*_*H*_ = *a*_*ij*_, increasing *t*_*H*_ afterward to target the next weak link in the network, until all links of the network were deleted. The global efficiency *G* was computed at each threshold, and the final *G*^PD^(*t*_*H*_) values stored for further analysis.

*Synaptic scaling degradation* (SSD). This model also progressively deleted the weakest output links, but with the crucial additional rule that, upon link removal (with weight *t*_*H*_), all surviving output links shared the weight of the removed one, increasing theirs proportionally (Figure 4A, right). Thus, if there were at least two links and the total output strength before deletion was *s*_*i*_^bef^ = ∑_*j*_ *a*_*ij*_^bef^, the new weights were given by *a*_*ij*_ = *a*_*ij*_^bef^ ⋅ *s*_*i*_^bef^/(*s*_*i*_^bef^ − *t*_*H*_). If only one output link was present, it was deleted as in PD. The new global efficiency of the network *G*^SSD^(*t*_*H*_) was then computed, the threshold updated to seek for the new weakest links, and the process started again. Simulation ended when all links in the network were removed, and the final *G*^SSD^(*t*_*H*_) values stored.

The *G*^PD^(*t*_*H*_) and *G*^SSD^(*t*_*H*_) data obtained from the models was finally treated as in the experiments. First, for each simulated network, the *t*_*H*_ values (which ranged from *a*_min_ to *a*_max_ in the PD model, and from *a*_min_ to a certain $a_{\text{max}}^{*}$ ≥ *a*_max_ for SSD) were scaled as *d* ∈ [0, 1]. The new *G*^PD^(*d*) and *G*^SSD^(*d*) curves were then scaled relative to their values at *d* = 0 and averaged among realizations to obtain the scaled global efficiencies G_EFF_(*d*), which could be directly compared with the experiments.

### Ethics Statement

All procedures were approved by the Ethical Committee for Animal Experimentation of the University of Barcelona, under order DMAH-5461, and in accordance to the regulations of the Generalitat de Catalunya (Spain).

## AUTHOR CONTRIBUTIONS

Estefanía Estévez-Priego: Data curation; Formal analysis; Investigation; Methodology; Software; Visualization; Writing - Original Draft. Sara Teller: Data curation; Methodology; Resources; Software; Supervision; Validation. Clara Granell: Data curation; Formal analysis; Methodology; Validation; Visualization; Writing - Review & Editing. Alex Arenas: Conceptualization; Formal analysis; Funding acquisition; Investigation; Project administration; Resources; Supervision; Writing - Review & Editing. Jordi Soriano: Conceptualization; Funding acquisition; Investigation; Methodology; Resources; Software; Supervision; Validation; Writing - Original Draft; Writing - Review & Editing.

## FUNDING INFORMATION

Jordi Soriano, Fundació Bancària “La Caixa” (http://dx.doi.org/10.13039/100000778), Award ID: LCF/PR/HR19/52160007. Jordi Soriano, H2020 Future and Emerging Technologies (http://dx.doi.org/10.13039/100010664), Award ID: 713140 MESOBRAIN. Estefanía Estévez-Priego, H2020 Future and Emerging Technologies (http://dx.doi.org/10.13039/100010664), Award ID: 713140 MESOBRAIN. Jordi Soriano, Secretaría de Estado de Investigación, Desarrollo e Innovación (http://dx.doi.org/10.13039/501100007136), Award ID: FIS2013-41144-P, FIS2016-78507-C2-2-P, FIS2017-90782-REDT. Sara Teller, Secretaría de Estado de Investigacíón, Desarrollo e Innovación (http://dx.doi.org/10.13039/501100007136), Award ID: FIS2013-41144-P, FIS2016-78507-C2-2-P, FIS2017-90782-REDT. Jordi Soriano, Departament d’Innovació, Universitats i Empresa, Generalitat de Catalunya (http://dx.doi.org/10.13039/501100002943), Award ID: 2017-SGR-1061. Alex Arenas, Departament d’Innovació, Universitats i Empresa, Generalitat de Catalunya (http://dx.doi.org/10.13039/501100002943), Award ID: 2017-SGR-896. Alex Arenas, Secretaría de Estado de Investigación, Desarrollo e Innovación (http://dx.doi.org/10.13039/501100007136), Award ID: PGC2018-094754-B-C21. Alex Arenas, Universitat Rovira i Virgili (http://dx.doi.org/10.13039/501100007512), Award ID: 2017PFRURV-B2-41. Alex Arenas, ICREA Academia. Alex Arenas, James S. McDonnell Foundation (http://dx.doi.org/10.13039/100000913), Award ID: 220020325. Clara Granell, James S. McDonnell Foundation (http://dx.doi.org/10.13039/100000913), Award ID: 220020457. Clara Granell, Secretara de Estado de Investigación, Desarrollo e Innovación (http://dx.doi.org/10.13039/501100007136), Award ID: Juan de la Cierva.

## TECHNICAL TERMS

Resilience: The capacity of a neuronal circuit to maintain activity and functional characteristics upon loss or weakening of neurons or connections.
Synaptic plasticity: The strengthening or weakening of synapses according to neuronal activity, for instance, to potentiate specific connectivity pathways.
Synaptic scaling: A regulatory mechanism of neuronal circuits by which synaptic strength is adjusted to compensate for chronic changes in activity.
Functional organization: A set of global network traits, such as modularity or global efficiency, that imprint distinct organizational characteristics to a network.
CNQX (6-cyano-7-nitroquinoxaline-2,3-dione): Antagonist of AMPA-glutamate receptors in excitatory neurons, frequently used to either weaken excitatory synaptic communication or block it entirely.
Global efficiency: A measure of network capacity for information exchange. The higher the overall connectivity among nodes, the higher the global efficiency.
Hyperefficiency: In the context of our work, an abrupt increase of global efficiency resulting from a strengthening of effective connections.
Percolation: Behavior of information or other measure as nodes or connections are removed, typically separ0061ting a ‘connected’ and a ‘disconnected’ phase.
Effective connectivity: Causal statistical relationship among the activity patterns of neurons in a network, leading to directed and weighted adjacency connectivity matrices.
Modularity: Tendency of the nodes in a network to connect within a group more strongly than with nodes in another group.
Calcium imaging: A noninvasive experimental technique in which neuronal activity is monitored through calcium-sensitive fluorescent dyes, either chemically loaded or genetically encoded.
